# Differences in carbon source usage by dental plaque in children with and without early childhood caries

**DOI:** 10.1038/ijos.2017.47

**Published:** 2017-12-20

**Authors:** Yan Zhao, Wen-Jie Zhong, Zhe Xun, Qian Zhang, Ye-Qing Song, Yun-Song Liu, Feng Chen

**Affiliations:** 1 The Second Dental Center, Peking University School and Hospital of Stomatology, Beijing 10081, China; 2 Central Laboratory, Peking University School and Hospital of Stomatology, National Engineering Laboratory for Digital and Material Technology of Stomatology, Beijing Key Laboratory of Digital Stomatology, Beijing 100081, China; 3 Department of Prosthodontics, Peking University School and Hospital of Stomatology, Beijing 10081, China

**Keywords:** Biolog assay, carbon source utilization, early childhood caries, microbial community

## Abstract

Early childhood caries (ECC) is a considerable pediatric and public health problem worldwide. Preceding studies have focused primarily on bacterial diversity at the taxonomic level. Although these studies have provided significant information regarding the connection between dental caries and oral microbiomes, further comprehension of this microbial community’s ecological relevance is limited. This study identified the carbon source metabolic differences in dental plaque between children with and without ECC. We compared the microbial community functional diversity in 18 caries-free subjects with 18 severe ECC patients based on sole carbon source usage using a Biolog assay. The anaerobic microbial community in the ECC patients displayed greater metabolic activity than that of the control group. Specific carbon source metabolism differed significantly between the two groups. Subjects from the two groups were well distinguished by cluster and principal component analyses based on discriminative carbon sources. Our results implied that the microbial functional diversity between the ECC patients and healthy subjects differed significantly. In addition, the Biolog assay furthered our understanding of oral microbiomes as a composite of functional abilities, thus enabling us to identify the ecologically relevant functional differences among oral microbial communities.

## Introduction

Early childhood caries (ECC), previously known as nursing bottle mouth and nursing caries, is a leading pediatric and public health problem in China and worldwide.^1^ It is the most common chronic disease in children and is increasing in prevalence among 2 to 5 year olds, with low consultation rates.^2^ Caries in young children are usually difficult and expensive to manage, and antibiotics, general anesthesia and hospital admission may be required for treatment.^3^

Many genetic, behavioral, social and environmental factors are involved in ECC etiology, supporting the disease’s multifactorial nature.^4–5^ Although many risk factors are associated with ECC, dental caries is a biofilm-dependent infectious disease, resulting from microbial interactions, host factors, time and diet (sugars), that modulate the dynamic biofilm formation on tooth surfaces.^6^ Dental biofilm consists of a bacterial ecosystem that exhibits various physiological characteristics. Acid production resulting from bacterial carbohydrate metabolism and the subsequent decrease in environmental pH are responsible for demineralizing the tooth surfaces. The transition in plaque composition between healthy and disease states is suggested to be driven by the microbial community response to environmental changes, resulting in the selection of previously minor microbial flora components.^7^ In addition, the same sucrose intake by different individuals or populations results in large disparities in caries severity.^8^ Individual oral environments and microbiota composition differ intrinsically, thus leading to individual differences in caries progression and treatment outcomes.^9–10^

Over the past few decades, extensive research has provided much information on the connection between dental caries and dental plaque bacteria.^11^ Dental caries-associated oral streptococci are referred to as the mutans streptococci, and *Streptococcus mutans* and *Streptococcus sobrinus* are the predominant caries-associated species in humans.^12^ The earlier that high oral mutans streptococci counts occur in infancy, the more severe the caries in the primary dentition.^13^ Mutans streptococci also exhibit higher prevalence and proportions in caries-positive subjects than in caries-free individuals.^14–15^

To date, few studies have investigated carbon source usage by the mixed oral microflora of caries. The Biolog assay was created specifically for community analysis and microbial ecological studies, and it uses a rapid, community-level physiological profile to assess patterns of sole carbon source use by mixed microbial samples.^16–17^

Despite the technological advances in microbial flora research, few studies have investigated caries microflora.^18^ Therefore, we investigated supragingival plaque samples to determine potential differences in carbon source usage between children with and without ECC to explore possible functional differences in the microbial flora.

## Materials and methods

### Ethical approval and informed consent

This study was approved by the Human Research Ethics Committee of Peking University School of Stomatology (PKUSSIRB-2012066).

### Subjects

Children seeking an examination or treatment at the Pediatric Dentistry Department of the Second Dental Center, Peking University School and Hospital of Stomatology were recruited using a computer-generated random number table from 1 January to 31 August 2014. All parents of the pediatric subjects provided written informed consent before study enrollment. Inclusion criteria were as follows: age range from 48 to 71 months; no infectious, congenital or periodontal diseases or dental abscesses; and no antibiotic use, fluoride therapy or tooth extraction during the month before dental plaque collection.

A single faculty member whose diagnostic criteria were calibrated with another experienced colleague performed all dental examinations. Examinations were performed with a mouth mirror and dental probe to detect caries through visual/tactile methods. Teeth were cleaned using a cotton pellet, and no radiographs were taken. We assessed decayed, missing and filled primary teeth (dmft) based on the dental caries diagnostic criteria of the World Health Organization (1997).^19^ Children with five or more decayed teeth were placed in the SECC group, and caries-free (CF) children were placed in the control group. Restored teeth with recurrent caries were considered decayed.^20–21^ Dental examinations were repeated on all subjects from August to October 2015. Children from the control group remained CF.

In total, this cross-sectional study enrolled 36 children, of whom 18 had severe ECC (SECC) and 18 were CF.

### Sample collection and processing

The subjects were asked not to eat more food or brush their teeth after breakfast on the morning of sample collection. Approximately 2 h after breakfast, they were asked to rinse their mouths with water. For each child, dental plaque was sampled from the intact enamel of the eight deciduous molars. The samples were collected using a sterile excavating spoon hand instrument and were immediately placed in an Eppendorf tube containing 1 mL of sodium thiosulfate solution. Samples were stored at 0 °C in an ice bag and sent to the laboratory within 2 h after collection.

### Biolog assay

The samples were resuspended in 11 mL of 0.01 mol·L^−1^ phosphate-buffered saline, pH 7.2–7.4, and vortexed thoroughly for 60 s. Ten samples from each group were inoculated into Biolog anaerobic-negative (AN) microplates (Biolog Inc., CA, USA) at 100 μL per well. The other eight samples from each group were inoculated into Biolog GEN III microplates (Biolog Inc., CA, USA) (for both Gram-negative and Gram-positive bacteria). Biolog AN microplates contain 95 separate carbon sources as shown in Supplementary Table S1, and a blank well with water only. GEN III microplates test for Gram-negative and Gram-positive bacteria in the same test panel. The test panel contains 71 carbon sources and 23 chemical sensitivity assays. Each well contains tetrazolium violet, and the color caused by the reduction reaction indicates respiration. Initial optical densities (ODs) of the plaque suspensions were measured before inoculation.

Plates were incubated in a 5% CO_2_ incubator at 37 °C for 4 days (Biolog AN plates were packed in anaerobic bags). The OD at 590 nm (OD_590nm_) in each well was recorded every 24 h using the Biolog MicroStation and associated software.

### Statistical analysis

To correct for background activity, the corrected OD value was introduced, which was obtained by subtracting the control well OD from the experimental well OD. If the difference was negative, the corrected OD value was deemed to be zero.

The overall metabolic activity for each microbial community in the Biolog AN plates was expressed as the average well color development (AWCD), which is the mean value of the corrected OD values in wells that contained sole carbon sources.

We standardized the corrected OD value by dividing each corrected OD value by the AWCD of each plate to avoid artificial differences from initial OD value variations in the plaque suspensions among microbial communities.

An independent *t*-test was performed to compare the mean differences in AWCD between the CF and SECC groups over four days. A *P<*0.05 or *P<*0.01 was considered statistically significant.

The sole carbon source distributions of the two groups were measured. Based on standardized OD values, differences in exploited carbon sources were measured using independent *t*-tests. The relationship between the CF and SECC groups based on discriminative carbon sources was determined using cluster analysis and principal component analyses (PCAs).

## Results

### Functional diversity in the CF and SECC groups

**Figure 1:**
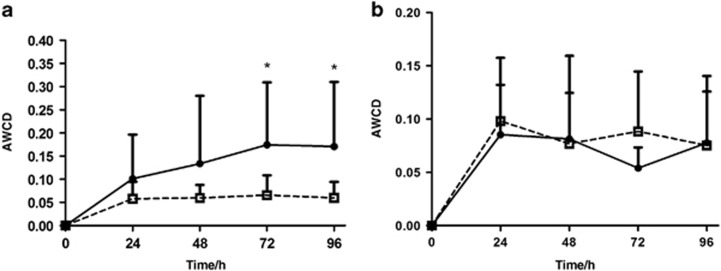
**AWCD with incubation time in the CF (**□**) and SECC (**•**) groups.** (**a**) Incubated in the Biolog AN plates. (**b**) Incubated in the Biolog GN III plates. *P<0.05. AN, anaerobic-negative; AWCD, average well color development; CF, caries free; OD, optical density; SECC, severe early childhood caries.

Subjects’ demographic and clinical characteristics are displayed in Table 1. The initial OD value of the inoculum was higher in the SECC group than in the CF group, indicating that the SECC patients had more accumulated microbial community biomass. In the Biolog AN plates, the changes in community metabolism over time were well demonstrated by the Biolog assay, reflecting the changes in community structure. During the first 48 h after inoculation, no significant difference in the overall AWCD was found between the CF and SECC groups. During later incubation periods, however, the SECC group yielded greater color responses, and significant differences were noted between the two groups (Figure 1a). In the Biolog GEN III plates, however, no significant difference was noted between the two groups (Figure 1b). Thus, the following results describe the samples cultivated in the Biolog AN plates, unless otherwise stated.

### Patterns of discriminative sole carbon source usage

**Figure 2:**
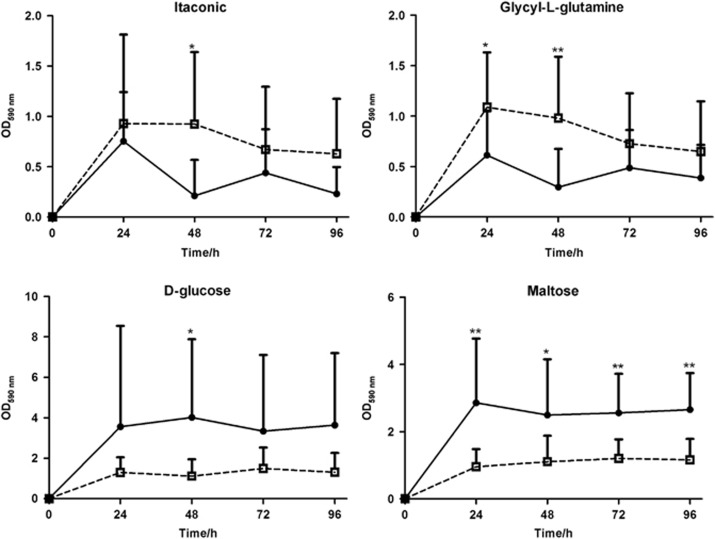
**Catabolic kinetics based on incubation time for discriminative positive carbon sources in the CF (**□**) and SECC (**•**) groups.** Two major patterns of carbon source usage were observed (*P<0.05 and **P<0.01). CF, caries free; OD, optical density; SECC, severe early childhood caries.

The analyses of 95 sole carbon source usage patterns showed 10, 23, 9 and 7 significantly different (*P*<0.05 or *P*<0.01) carbon sources between the two groups at 24, 48, 72 and 96 h, respectively. Additional data are provided in Supplementary Table S2. Of these, two major patterns were detected (Figure 2). The discriminative carbons sources detected in the GEN III system are also listed in Supplementary Table S1.

**Figure 3:**
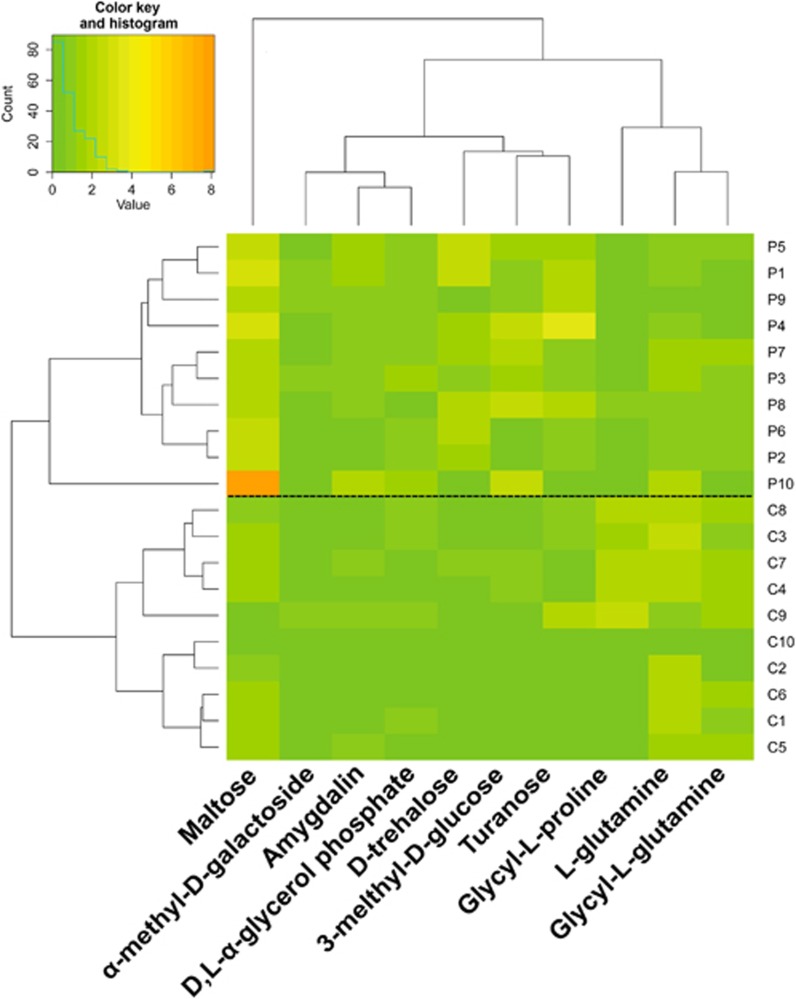
**Cluster analyses of 20 clinical plaque samples from the caries-free control (C) group and the caries patient (P) group based on standardized OD values of the discriminative carbon sources at 24 h.** OD, optical density.

Cluster analyses of the 20 subjects from each group were performed based on the 10 carbon sources used differently at 24 h (Figure 3) to identify correlations between the carbon source consumption pattern and health status. The results indicated that the 20 plaque samples could be classified into two clusters, which coincided exactly with the clinical classification based on diagnosis. Among the 10 discriminative carbon sources, the consumption patterns were classified into two groups: maltose as cluster 1 and the other nine sources as cluster 2, where the consumption pattern of the six carbohydrates was clearly distinguished from that of the three amino acids and peptides.

**Figure 4:**
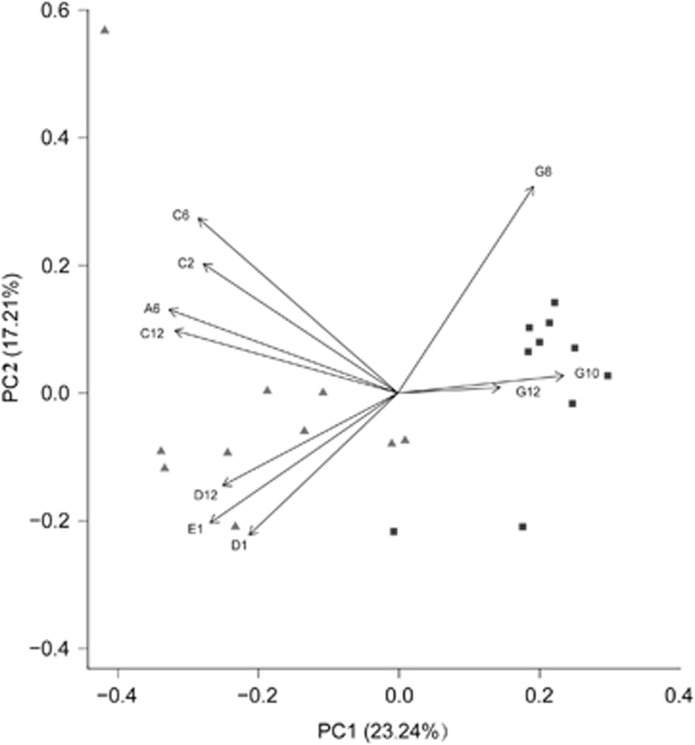
**Ordination biplot of principal component analyses of the carbon source usage patterns of the microbial communities in the CF (**▪**) and SECC (**▴**) groups.** Arrows indicate the directions and relative importance (arrow lengths) of the 10 substrate variables. CF, caries free; OD, optical density; PC, principal component; SECC, severe early childhood caries.

The usage patterns of each microbial community were compared using PCAs of the 24-h absorption data. Microbial community samples from the 20 subjects served as the objects, and the absorbance values of discriminative carbon source usage were the variables (Figure 4). CF and SECC group samples showed distinct sole carbon source patterns. CF microbial community samples had lower coordinated values (PC scores) for PC1, which explained 23.24% of the data variance compared with the SECC samples. The second PC explained 17.21% of the variance.

Analysis of PC1 showed that SECC microbial communities used several carbohydrates and sugars (amygdalin, D,L-α-glycerol phosphate, maltose, 3-methyl-L-glucose, α-methyl-L-galactoside, L-trehalose and turanose) to a greater degree than the CF microbial communities, while the latter showed greater relative use of the three amino acids and peptides (L-glutamine, glycyl-L-glutamine and glycyl-L-proline).

## Discussion

This study explored the possible functional differences in microbial flora between children with and without ECC. We compared the dental biofilm functional diversity based on sole carbon source usage using a Biolog assay. Greater metabolic activity was observed in the SECC patients’ anaerobic microbial community compared to that of the control group. We also identified significant differences in specific carbon source use between the two groups. Subjects from the two groups were clearly distinguished by cluster and principal component analyses based on discriminative carbon sources.

The Biolog assay demonstrated its fundamental utility for environmental microbial community studies, with respect to their functional potential. Other studies have used Biolog microplates to detect metabolic changes in polluted environmental microbial communities^22^ and to evaluate ecological environmental restoration effects.^23^ Apart from these technological advances in microbial flora research, attempts have been made to expand Biolog microplate use to the oral microbial flora. Zhang *et al.*^17^ previously used this tool to describe functional diversity based on carbon source use in microbial communities from healthy subjects and periodontitis patients.

The Biolog assay has its limitations, however. For example, it cannot detect microorganisms that do not use the carbon sources on the Biolog microplate, and the response to substrate catabolism requires effective quantity and activity of the microbial community in the tested samples.

The results of our analysis that SECC microbial communities used more carbohydrates than the control group led us to investigate the corresponding change in microbial profiles. Xu *et al.*^24^ used pyrosequencing to elucidate supragingival plaque bacterial diversity between ECC patients and healthy children with unerupted second primary molars. They found that *Streptococcus* represented more of the total flora of children in the ECC group than in the caries-free group, which was shown to be one of the main reasons for the plaque flora distinction between the two groups. Genome findings and current knowledge of *Streptococcus mutans* (MS) sugar metabolism suggest that it metabolizes a wider variety of carbohydrates than any other Gram-positive organism sequenced to date.^25–26^

Caries risk assessment is beneficial for controlling dental caries and preventing their development.^27^ One caries activity test counts the *Streptococcus mutans* (MS) or *Lactobacilli* colony-forming units using selective growth media that allows for growth of the specific cariogenic bacterial colonies that contribute to the initiation and progression of dental caries.^28^ This is because MS is believed to be the main etiological agent of dental caries due to its sugar-fermenting and acidogenic characteristics. Most diagnostic, preventive and therapeutic strategies have consequently targeted this microorganism.^29–30^

However, as per recent studies on caries etiology, caries are caused by the collective, and likely synergistic, interactions among multiple microorganisms in the dental plaque, which has been confirmed by DNA- and RNA-based studies of carious lesions.^31^ Because caries microbiology does not have a unique etiology, focusing on the disease-associated metabolic characteristics of microbial communities, for example, the shifting abundance of specific metabolic modules/pathways, rather than determining the specific causative agents, may help us to better understand and control the disease. A recent study assessed adenosine triphosphate activity in dental plaque and MS cultures to evaluate caries activity but found both indicators were not significantly associated with caries activity at 24 months.^32^

In this study, we measured dmft based on the dental caries diagnostic criteria defined in the WHO Basic Methods (1997); however, a recently developed International Caries Detection and Assessment System (ICDAS-II) can be used in epidemiological studies, public health research, clinical research, clinical practice and dental education. Evidence shows that ICDAS-II is reproducible and accurately detects occlusal and proximal caries, especially non-cavitated caries.^33–34^ Thus, ICDAS-II criteria are a promising tool for early caries diagnosis, and we will adopt it in our further studies.

In our analysis, significant differences in AWCD between the two groups were detected in the anaerobic, rather than the aerobic, microbial community. This indicates that anaerobic microbiota may contribute significantly to ECC pathogenesis. Comparing our results with a previous study concerning periodontitis revealed etiological differences in these two common oral diseases. The initial OD values were higher in both diseased groups than in the corresponding healthy groups. Significant differences in the AWCD between the healthy and periodontitis groups were noted during the first 24 h of inoculation, indicating shifts in both the biomass and composition of the microbial community in periodontitis patients. However, for caries, no significant difference in the AWCD of the Biolog plates was found until later in the incubation, which may reflect the different etiologies between caries and periodontitis. Differential carbon source use differentiated healthy subjects from ECC patients, and this indicated that the active microbiotas responsible for the two diseases are different.

Both the cluster analyses and PCA were conducted based on discriminative carbon source usage at 24 h, which is closest to the *in situ* functions of the plaque samples. The resulting clusters further improved the metabolic discrimination of specific carbon sources between the CF and SECC groups, suggesting that caries status can be distinguished using Biolog AN microplates. We also believe that this method is a potential prognostic tool for future disease diagnosis in the absence of clinical symptoms.

It is essential to further investigate discriminative carbon sources at the category level. PCA revealed the carbon sources that contributed most to diversity. PC1 analysis showed that microbial communities from the SECC group used several carbohydrates and sugars (amygdalin, L,L-α-glycerol phosphate, maltose, 3-methyl-L-glucose, α-methyl-L-galactoside, L-trehalose and turanose) to a greater degree than the CF microbial communities. This supports current knowledge of the relationship between dietary sugars and caries. Dietary sugars are one of the most important mediators in ECC pathogenesis because they are the bacteria's primary energy source and substrate for acid and exopolysaccharides. In addition, CF subjects’ microbiota showed greater relative use of the three amino acids and peptides (L-glutamine, glycyl-L-glutamine and glycyl-L-proline). No significant differences in nucleotide and nucleoside usage were noticed between the two groups.

Apart from the importance of bacteriology in dental caries etiology, studies have reported that the eating habits and socioeconomic status of children and their parents or caregivers are good ECC predictors.^35^ Detecting inappropriate habits in an interview can help dentists obtain early indications and establish a targeted health education program regarding diet. In addition, oral health promotion programs based on repeated preventive guidance initiated during the mother’s pregnancy were successful in reducing the incidence of severe ECC in young children.^36^ In our study, we revealed specific carbohydrates that were more commonly used in the SECC group, and we speculate that these carbohydrates should be avoided in the diet to prevent susceptible individuals from developing ECC.

One limitation of this study is the insufficient sample size. We referred to our previous work^17^ when we determined the sample size because few studies have investigated carbon source use by the mixed oral microflora. More subjects of varying disease severity should be included in future studies. Furthermore, the incubated condition of the Biolog assay cannot fully simulate the real oral environment. Microbial growth is important in this assay; thus, metabolic patterns exhibited here reflected functional potential rather than *in situ* functional ability.

Our results implied a significant difference in microbial functional diversity between children with and without ECC. The Biolog assay shows potential for identifying the ecologically relevant functional differences among oral microbial communities. Increasing evidence shows that in healthy subjects or patients with specific disease conditions, the microbiome composition may fluctuate or even change drastically over time, but its functional profile remains relatively stable. This suggests that the relative abundance pattern of metabolic modules/pathways may be indicative of healthy or certain diseased conditions. Our results support this view, and future longitudinal studies should be conducted to clarify the association between microbial pathways and disease.

## Figures and Tables

**Table 1:** Demographic and clinical characteristics of the subjects

Items	CF group	SECC group
Sample size	18	18
Gender (male/female)	9/9	9/9
Age (months±s.d.)	62.0±8.4	57.9±8.7
Decayed, missing and filled primary teeth (dmft)	0	7.8±2.1

Subject demographics and clinical characteristics.

CF, caries free; SECC, severe early childhood caries.
