# Supplementary material for: Differences in carbon source usage by dental plaque in children with and without early childhood caries
Source: Int J Oral Sci. 2017 Dec 20;9(12):e6–. doi: 10.1038/ijos.2017.47 (PMC5750455; doi:10.1038/ijos.2017.47)
Supplement: Supplementary Table S1 [file ijos201747x1.pdf]

Table S1 (a) Carbon-sources pattern of the Biolog AN microplate. Colors show the category of each well: yellow stands for carbohydrates and sugar; orange stands for organic acids; green stands for amino acids, peptides and related cheminals; and blue stands for nucleotides and nucleoside.

A1<br>Water	A2<br>N-Acetyl-D-Galactosamine	A3<br>N-Acetyl-D-Glucosamine	A4<br>N-Acetyl-β-D-Mannosamine	A5<br>Adonitol	A6<br>Amygdalin	A7<br>D-Arabitol	A8<br>Arbutin	A9<br>D-Cellobiose	A10<br>α-Cyclodextrin	A11<br>ß-Cyclodextrin	A12<br>Dextrin
B1<br>Dulcitol	B2<br>i-Erythritol	B3<br>D-Fructose	B4<br>L-Fucose	B5<br>D-Galactose	B6<br>D-Galacturonic Acid	B7<br>Gentiobiose	B8<br>D-Gluconic Acid	B9<br>D-Glucosaminic Acid	B10<br>α-D-Glucose	B11<br>Glucose-1-Phosphate	B12<br>Glucose-6-Phosphate
C1<br>Glycerol	C2<br>D,L-α-Glycerol Phosphate	C3<br>m-Inositol	C4<br>α-D-Lactose	C5<br>Lactulose	C6<br>Maltose	C7<br>Maltotriose	C8<br>D-Mannitol	C9<br>D-Mannose	C10<br>D-Melezitose	C11<br>D-Melibiose	C12<br>3-Melthyl-D-Glucose
D1<br>α-Methyl-D-Galactoside	D2<br>ß-Methyl-D-Galactoside	D3<br>α-Methyl-D- Glucoside	D4<br>ß-Methyl-D-Glucoside	D5<br>Palatinose	D6<br>D-Raffinose	D7<br>L-Rhamnose	D8<br>Salicin	D9<br>D-Sorbitol	D10<br>Stachyose	D11<br>Sucrose	D12<br>D-Trehalose
E1<br>Turanose	E2<br>Acetic Acid	E3<br>Formic Acid	E4<br>Fumaric Acid	E5<br>Glyoxylic Acid	E6<br>α-Hydroxybutyric Acid	E7<br>ß-Hydroxybutyric Acid	E8<br>Itaconic	E9<br>α-Ketobutyric Acid	E10<br>α-Ketovaleric Acid	E11<br>D,L-Lactic Acid	E12<br>L-Lactic Acid
F1<br>D-Lactic Acid Methyl Ester	F2<br>D-Malic Acid	F3<br>L-Malic Acid	F4<br>Propionic Acid	F5<br>Pyruvic Acid	F6<br>Pyruvic Acid Methyl Ester	F7<br>D-Saccharic Acid	F8<br>Succinamic Acid	F9<br>α-Succinic Acid	F10<br>Succinic Acid Mono-Methyl Ester	F11<br>m-Tartaric Acid	F12<br>Urocanic Acid
G1<br>L-Alaninamide	G2<br>L-Alanine	G3<br>L-Alanyl-L-Glutamine	G4<br>L-Alanyl-L-histidine	G5<br>L-Alanyl-L-Threonine	G6<br>L-Asparagine	G7<br>L-Glutamic Acid	G8<br>L-Glutamine	G9<br>Glycyl-L-Aspartic Acid	G10<br>Glycyl-L-Glutamine	G11<br>Glycyl-L-methionine	G12<br>Glycyl-L-Proline
H1<br>L-Methionine	H2<br>L-Phenylalanine	H3<br>L-Serine	H4<br>L-Threonine	H5<br>L-Valine	H6<br>L-Valine plus L-Aspartic Acid	H7<br>2’-Deoxy Adenosine	H8<br>Inosine	H9<br>Thymidine	H10<br>Uridine	H11<br>Thymidine-5’-Monophosphate	H12<br>Uridine-5’-Monophosphate

Table S1 (b) Carbon-sources pattern of the Biolog GEN III microplate. The test panel contains 71 carbon sources (columns 1-9) and 23 chemical sensitivity assays (columns 10-12).

A1<br>Negative Control	A2<br>Dextrin	A3<br>D-Maltose	A4<br>D-Trehalose	A5<br>D-Cellobiose	A6<br>Gentiobiose	A7<br>Sucrose	A8<br>Turanose	A9<br>Stachyose	A10<br>Poitive Control	A11<br>pH6	A12<br>pH5
B1<br>D-Raffinose	B2<br>α-D-Lactose	B3<br>D-Melibiose	B4<br>ß-Methyl-D-Glucoside	B5<br>Salicim	B6<br>N-Acetyl-D-Glucosamine	B7<br>N-Acetyl-ß-D-Mennosamine	B8<br>N-Acetyl-D-Galacetosamine	B9<br>N-AcetylNeuramimic acid	B10<br>1% NaCl	B11<br>4% NaCl	B12<br>8% NaCl
C1<br>α-D-Glucose	C2<br>D-Mannose	C3<br>D-Fructose	C4<br>D-Galactose	C5<br>3-Methyl-D-Glucose	C6<br>L-Fucose	C7<br>D-Fucose	C8<br>L-Rhamnose	C9<br>Imosine	C10<br>1% Sodium Lactate	C11<br>Fusidic Acid	C12<br>D-Serine
D1<br>D-Sorbitol	D2<br>D-Mannitol	D3<br>D-Arabitol	D4<br>myo-Inositol	D5<br>Glycerol	D6<br>D-Glucose-6-PO4	D7<br>D-Fructose-6-PO4	D8<br>D-Aspartic Acid	D9<br>D-Serine	D10<br>Troleandomycin	D11<br>Rifamycin SV	D12<br>Minocycline
E1<br>Gelatin	E2<br>Glycyl-L-Prolin	E3<br>L-Alanine	E4<br>L-Arginine	E5<br>L-Aspartic Acid	E6<br>L-Glutamic Acid	E7<br>L-Histidine	E8<br>L-Pyroglutamic Acid	E9<br>L-Serine	E10<br>Lincomycin	E11<br>Guanidine HCl	E12<br>Niaproof 4
F1<br>Pectin	F2<br>D-Galacturonic Acid	F3<br>L-Galactonic Acid Lactone	F4<br>D-Gluconic Acid	F5<br>D-Glucuronic Acid	F6<br>Glucuronamide	F7<br>Mucic Acid	F8<br>Quinic Acid	F9<br>D-Saccharic Acid	F10<br>Vancomycin	F11<br>Tetrazolium Violet	F12<br>Tetrazolium Blue
G1<br>p-Hydroxy-Phenylacetic Acid	G2<br>Methyl Pyruvate	G3<br>D-Lactic Acid Methyl	G4<br>L-Lactic Acid	G5<br>Citric Acid	G6<br>α-Keto-Glutaric Acid	G7<br>D-Malic Acid	G8<br>L-Malic Acid	G9<br>Bromo-Succinic Acid	G10<br>Nalidixic Acid	G11<br>Lithium Chloride	G12<br>Potassium Tellurite
H1<br>Tween 40	H2<br>γ-Amino-ButyricAcid	H3<br>α-Hydroxy-Butyric Acid	H4<br>β-Hydroxy-D,LButyric Acid	H5<br>α-Keto-Butyric Acid	H6<br>Acetoacetic Acid	H7<br>Propionic Acid	H8<br>Acetic Acid	H9<br>Formic Acid	H10<br>Aztreonam	H11<br>Sodium Butyrate	H12<br>Sodium Bromate
