# Supplementary material for: Differences in carbon source usage by dental plaque in children with and without early childhood caries
Source: Int J Oral Sci. 2017 Dec 20;9(12):e6–. doi: 10.1038/ijos.2017.47 (PMC5750455; doi:10.1038/ijos.2017.47)
Supplement: Supplementary Table S2 [file ijos201747x2.pdf]

Table S2 Discriminative carbon sources between the caries free (CF) group and severe early childhood caries (SECC) group based on standardized OD values. ↑: the utilization is significantly higher than the other group ($p < 0.05$), and ↑↑: $p < 0.01$.

(a) Biolog AN microplates.

Categories	Carbon sources	24 h		48 h		72 h		96 h	
		CF	SECC	CF	SECC	CF	SECC	CF	SECC
Carbohydrates and Sugar	N-Acetyl-D-Glucosamine				↑↑				↑
	Amygdalin		↑		↑				
	Arbutin						↑		
	i-Erythritol							↑	
	D-Fructose						↑		↑
	D-Galacturonic Acid						↑		
	α-D-Glucose				↑				
	D,L-α-Glycerol Phosphate		↑						

	α-D-Lactose			↑	↑	↑
	Maltose	↑↑		↑	↑↑	↑↑
	D-Mannose			↑		
	D-Melibiose			↑		
	3-Melthyl-D-Glucose	↑↑				
	α-Methyl-D-Galactoside	↑				
	Stachyose			↑	↑	↑
	D-Trehalose	↑↑		↑		
	Turanose	↑				
Organic Acids	Formic Acid		↑			
	Fumaric Acid		↑↑			
	Glyoxylic Acid		↑↑			
	α-Hydroxybutyric Acid		↑			

	ß-Hydroxybutyric Acid		↑		
	Itaconic		↑		
	D-Malic Acid		↑		
	Propionic Acid		↑		
	Succinamic Acid		↑		
	Urocanic Acid			↑	
Amino Acids, Peptides and Related Cheminals	L-Alanyl-L-Glutamine		↑		
	L-Alanyl-L-Threonine		↑↑		
	L-Glutamic Acid		↑		
	L-Glutamine	↑			
	Glycyl-L-Aspartic Acid		↑		↑
	Glycyl-L-Glutamine	↑	↑↑		
	Glycyl-L-Proline	↑		↑	

	L-Valine plus L-Aspartic Acid					↑			

(b) Biolog GN III microplates

Categories	Carbon sources	24 h		48 h		72 h		96 h	
		CF	SECC	CF	SECC	CF	SECC	CF	SECC
Carbohydrates and Sugar	N-Acetyl-D-Galactosamine					↑			
	D-Fucose					↑↑			
	L-Fucose					↑			
	L-Rhamnose					↑↑			
	L-Rhamnose					↑			
	D-Gluconic Acid		↑↑				↑		
Organic Acids	α-Keto-Butyric Acid				↑				
